# Decision Making in the PICU: An Examination of Factors Influencing Participation Decisions in Phase III Randomized Clinical Trials

**DOI:** 10.1155/2014/676023

**Published:** 2014-08-04

**Authors:** Laura E. Slosky, Marilyn Stern, Natasha L. Burke, Laura A. Siminoff

**Affiliations:** ^1^Division of Developmental and Behavioral Sciences, Children's Mercy Hospital, 2401 Gillham Road, Kansas City, MO 64108, USA; ^2^Department of Rehabilitation and Mental Health Counseling, University of South Florida, 13301 Bruce B. Downs Boulevard, MHC 1632, P.O. Box 12, Tampa, FL 33612, USA; ^3^Department of Psychology, University of South Florida, 4202 East Fowler Avenue, PCD4118G, Tampa, FL 33620, USA; ^4^Department of Social and Behavioral Health, Virginia Commonwealth University, P.O. Box 980149, Richmond, VA 23298, USA

## Abstract

*Background*. In stressful situations, decision making processes related to informed consent may be compromised. Given the profound levels of distress that surrogates of children in pediatric intensive care units (PICU) experience, it is important to understand what factors may be influencing the decision making process beyond the informed consent. The purpose of this study was to evaluate the role of clinician influence and other factors on decision making regarding participation in a randomized clinical trial (RCT). *Method*. Participants were 76 children under sedation in a PICU and their surrogate decision makers. Measures included the Post Decision Clinician Survey, observer checklist, and post-decision interview. *Results*. Age of the pediatric patient was related to participation decisions in the RCT such that older children were more likely to be enrolled. Mentioning the sponsoring institution was associated with declining to participate in the RCT. Type of health care provider and overt recommendations to participate were not related to enrollment. *Conclusion*. Decisions to participate in research by surrogates of children in the PICU appear to relate to child demographics and subtleties in communication; however, no modifiable characteristics were related to increased participation, indicating that the informed consent process may not be compromised in this population.

## 1. Introduction

Obtaining informed consent prior to subject participation in an experimental protocol is vital to maintain ethical standards and ensure respect for persons. Even with a number of national and international guidelines to ensure true informed consent [1–5], some in the field believe the concept is an elusive ideal [6]. Patients who participated in clinical trials were unaware of the particulars of the research (e.g., randomization, treatment arms, etc.) or that the treatment was experimental in nature [7–9], and information presented in the consent form was not always taken into account when making medical decisions [10]. Moreover, for nearly two-thirds of those approached for study participation, the consent form played no part in their participation decision [10].

Problems with comprehension and readability [11], a misconception of direct therapeutic benefit [9, 12], and not recognizing the ability to discontinue [9] or opt out of treatment [13] also impede the function of the informed consent process. Importantly, the decision making process involving informed consent may be flawed, especially in high pressure environments [14]. Given the profound and sometimes clinical levels of distress that surrogates (i.e., parents or legal guardians) of children in pediatric intensive care units (PICU) experience [15], it is important to understand what factors may be influencing the decision making process beyond the informed consent process. Properly addressing such factors would help protect the integrity of the informed consent process.

Although the informed consent process precludes coercion and denial of services, it may nevertheless be challenging for physicians (i.e., medical doctors) to fully disengage from the role of health care provider during the informed consent process. Physicians have significant influence on medical decisions that are made by their patients [16–18], and communication with the physician during clinical encounters is an important factor in the final decision to participate in clinical trials [19–21]. In a study involving oncology patients, 68% of the time physicians recommended that their patients participate in a clinical trial, which was related to the resultant decision to participate [22]. Surrogates often rely on their child's physicians for their knowledge and expertise in the medical care of their child [16, 17]. Therefore, it is hypothesized that treating physicians play a significant role in the decisions that surrogates make regarding research trial participation. Based on previous findings [23], it is expected that physicians recommending these research opportunities have a significant influence on their patients' decisions to participate in clinical trials.

The PICU is a multidisciplinary environment, and although the patient's physician may present trial information to eligible study participants, other health care providers (e.g., nurses and respiratory therapists) may also serve in this role. Clinician understanding and actual or perceived endorsement of the trial being presented [24, 25] and potential benefits to the patient are associated with participation decisions [24]. A personal physician may be more apt to handle these types of questions depending on the type of study and clinician training [24], and it is possible that a physician's explanation may have a stronger impact on decision to participate than another health care provider (HCP). Lastly, distrust of sponsoring institutions has been noted as a reason for declining participation in research studies [3, 26]. Therefore, mention of the sponsoring institution to potential participants will also be examined as a potential factor in the surrogate decision-making process.

This study provides insight into how influential physician recommendations are in this unique population of surrogate decision makers for critically ill children in potentially life threatening situations. It also provides insight into the role of different HCPs and additional putative factors in the decision-making process. There are three primary hypotheses. First, it is expected that a HCP's overt recommendation to participate in the RCT will be related to participation decisions such that surrogates will be more likely to enroll their child into the RCT. Second, it is expected that surrogates receiving RCT information from a physician will be more likely to enroll their child in the RCT than those receiving the information from another HCP. Third, it is expected that surrogates who are given information about the institution supporting the RCT will be more likely to enroll their child in the protocol than those not receiving this information.

## 2. Method

### 2.1. Participants

Participants were recruited from Rainbow Babies and Children's Hospital Pediatric Intensive Care Unit. Data were collected from a total of 76 surrogate decision makers of hospitalized children in the PICU, with each surrogate representing one pediatric case (see Table 1). Hospitalized children had suffered a wide range of illnesses and accidents and were all in need of mechanical ventilation for at least a 24-hour period.

### 2.2. Procedure

This study was part of a larger study on informed consent. On call research nurses were contacted once a child became eligible to participate in a greater than minimal risk phase III clinical trial. This individual first provided an explanation of the present study on informed consent and obtained written informed consent. Surrogates then participated in an informed consent conference regarding participation in a drug RCT with their child's HCP. In this conference, the HCP presented an opportunity to participate in a clinical trial to test the efficacy of an experimental pharmaceutical treatment. Randomization procedures were explained, safety concerns addressed, and voluntary participation stressed. If the surrogates expressed an interest in enrolling their child, they were given a consent form to read and provided written informed consent for the drug RCT. Some HCPs presenting trial information were study investigators and others were not. HCP involvement in the RCT presented was controlled for in study analyses. Conferences with surrogates were observed and audio-recorded by research assistants. Following this conference, surrogates participated in a private structured interview about this HCP interaction. Both the drug RCT and the current informed consent study were approved by University Internal Review Boards.

### 2.3. Measures

#### 2.3.1. Semistructured Post-Decision Interview

A brief interview was conducted with the surrogates after they finished the informed consent conference. The interview was conducted in private, without the child's HCP present. The purpose of this interview was to obtain participant specific characteristics and to assess understanding of the clinical trial and treatment options. Participant specific characteristics included decision making preferences, risk taking inclinations, quality of relationship with their HCP, subject assessment of treatment risks and benefits, comprehension of trial participation requirements, their final decision regarding participation in the clinical trial, and the rationale behind their decision.

#### 2.3.2. Post-Decision Clinician Survey

The HCP that participated in the informed consent conference completed a survey after the informed consent conference. This measure was used to gather information about the HCP and his or her level of involvement in the randomized clinical trial. Demographic data for the HCP was recorded and included type of HCP, specialty, board certification, membership in clinical trials groups, exposure to trials during training, whether or not the HCP was an investigator in the drug trial, age, gender, and race. The HCP also provided their perception of the strength of their recommendation regarding participation in the RCT and the likelihood of the patient to benefit from the clinical trial.

#### 2.3.3. The Observer Checklist

This instrument was developed for the current study and was used to document and assess the interactions between surrogates and their child's HCP. The checklist was completed by research assistants who observed the informed consent process; the audiotape was used for coding purposes. The measure lists specific information categories related to informed consent that were dichotomously coded as occurring or not occurring. Examples of information coded included whether the informed consent process included an explanation of risks, benefits, funding, and alternatives. Content initiators were also coded in relation to each topic area raised. Past studies achieved between 88 and 93% agreement on this measure among independent raters [14, 21], and the current study achieved an interrater reliability within this range at approximately 90% agreement. The measure was developed and chosen to capture unique aspects and interactions of the consent process not addressed by previously validated measures.

### 2.4. Statistical Analyses

A series of Chi-square analyses and *t*-tests were conducted to identify demographic factors significantly associated with the decision to participate in the RCT. Hierarchical logistic regression analyses were performed to test the primary hypotheses. Demographic factors associated with the decision to participate in the RCT were entered as control variables in the first step of the regression analyses followed by the specific research question (i.e., HCP's verbal recommendation to participate (coded as yes or no), HCP presenting trial information (coded as physician or other), or HCP mention of the sponsoring institution (coded as yes or no)). Each regression was then compared to a constant-only model to determine whether the proposed hypothesis was statistically significant. Effect sizes were analyzed with Cox and Snell *R*-square and Nagelkerke *R*-square. All analyses were performed using the statistical package for the social sciences (SPSS) version 16.0.

## 3. Results

Participation in the RCT was not associated with any surrogate level data (see Table 2). However, participation was associated with patient age such that surrogates of older children (*M*_AGE_ = 6.212) were more likely to consent to participate than surrogates of younger children (*M*_AGE_ = 3.055). Patient age was controlled for in study analyses.

For the first hypothesis, it was hypothesized that if a HCP gave a verbal statement to the surrogate decision maker advocating enrollment in the RCT, the surrogate would be more likely to enroll their child in the RCT. The model with age and HCP recommendation was not statistically different from the constant-only model, and the effect size of HCP recommendation was small (see Table 3). Since the results were contrary to previous findings, the model was evaluated without controlling for the age of the pediatric patient. This model was compared to a constant only model, and the results were still not significant, *X* ^2^(1) = .14, *P* > .05. The effect size of physician recommendation was even smaller, with Cox and Snell *R*-square = .002 and Nagelkerke *R*-square = .003. However, a post hoc correlation was used to examine the relationship between strength of HCP recommendation and the decision to enroll in the RCT, and the results were significant. The stronger the recommendation, the less likely the surrogate was to enroll their child; *r* = −.33, *P* < .05.

For the second hypothesis, it was hypothesized that the type of HCP presenting the trial to the surrogate decision maker would predict trial participation such that those receiving the information from a physician would be more likely to participate than those being informed by another type of HCP (e.g., nurse or respiratory therapist). The model with age and the HCP presenting the trial was not statistically different from the constant-only model, and the effect size of the HCP presenting the trial was small (see Table 3).

Lastly, it was hypothesized that if a clinician referenced the name of the institution supporting the trial and indicated that the study was approved by the institution's Internal Review Board, the surrogate would be more likely to enroll their child in the experimental protocol. The model with age and mention of the sponsoring institution was significantly different from the constant-only model, and the effect size of mentioning the sponsoring institution was moderate (see Table 3). The change in odds associated with a one-unit change in reference to the supporting institution was −2.30, indicating that if the supporting institution was mentioned, the surrogate was 2.30 times less likely to enroll their child in the experimental protocol.

## 4. Discussion

The purpose of the current study was to elucidate factors that influence surrogate decision makers in the PICU to participate in RCTs. This is particularly important given the informed consent process has previously been found to be flawed, particularly in stressful environments [14]. It is therefore important to understand what may be influencing such decisions in this vulnerable population. Demographic variables were analyzed as potential covariates and putative factors predicting trial participation were analyzed including explicit verbal recommendation by the HCP to participate, the type of HCP presenting the trial, and HCP mention of the institution supporting the trial. Results from the majority of demographic variables were not significant; those who chose to participate in the RCT were not significantly different from those who elected standard treatment on most demographic domains. However, age of the pediatric patient was related to surrogates' decisions to enroll their child in the RCT. Surrogates who had an older child in the PICU were more likely to enroll them in the RCT than those with a younger child. A younger child in the PICU may be perceived as more vulnerable and unable to handle treatment when little is known about its safety and side effects. Younger children generally are seen as more vulnerable and less able to handle both adverse and normative events; however, this view may or may not be well founded [27, 28]. Nevertheless, patient age was controlled for in study analyses.

The relationship between an explicit HCP recommendation to participate in the RCT and the decision to participate was not significant, which indicates that a HCP recommending participation in the RCT did not predict whether a surrogate enrolled their child in the RCT. This was contrary to previous research findings, so the analysis was repeated without controlling for the age of the pediatric patient. The model still did not achieve statistical significance after age was excluded indicating that age of the pediatric patient was not accounting for the nonsignificant relationship. A larger sample size may be needed to detect a significant effect of physician recommendation on participation. However, it is possible that the levels of extreme stress that are unique to this time pressured situation may affect decision making in a manner differently than previously hypothesized, particularly in this population of surrogates that has children with acute complications of chronic conditions. Stress affects brain regions that are responsible for complex cognitive processes [29]. This potential change in cognitive functioning may cause surrogate decision makers to make decisions regarding clinical trial participation before the information is presented to them. In fact, previous research has found that this decision prior to information presentation is one of the strongest predictors of participation [30]. This type of informed consent study has also never been conducted in such a high stress environment or with such a fragile population. These changes in environmental stress and potential harm to the patient may be more salient when making decisions related to decision making regarding research participation. Further, participants had already agreed to participate in a study of informed consent which likely impacted their thought processes surrounding drug trial participation which is also likely to play a contributory role in current findings. Overall, however, the HCP's endorsement not affecting participant enrollment is a positive outcome and consistent with the ultimate goals of the informed consent process.

No relationship was found between the type of HCP presenting trial information and a surrogate's decision to participate. This indicates that a physician presenting the RCT to the surrogate did not reliably predict trial participation over another type of HCP presenting the same information. This implies that one type of clinician does not have more persuasive power than another, regardless of rank and role in the child's care. This has important implications for the informed consent process and supports the ultimate goal of this construct, protecting the patient. If a given individual had more persuasive power in these acutely stressful situations, they could more easily manipulate the outcome [31, 32]. This would present a significant conflict of interest within clinical practice, which could become especially problematic if the presenting clinician was a primary investigator for the trial desiring to recruit participants. The possible conflict of interest would have the potential to compromise patient care and the best interests of the child. However, as this study has revealed, the type of HCP presenting does not impact the resultant decision to participate. It therefore appears that this aspect of protection of human subjects is not compromised.

The third hypothesis examined the relationship between clinician mention of the institution sponsoring the RCT and participation. The results indicated that the mention of the institution supporting the trial reliably distinguished between surrogates enrolling their child in the experimental protocol from those surrogates who did not enroll their child. While this predictive relationship was significant, the direction of the relationship was unexpected. Specifically, analyses revealed that when the institution was mentioned, surrogates were less likely to enroll their child in the experimental protocol. This finding suggests that mention of the supporting institution may remind the surrogate of the experimental nature of the protocol, leading to a lower participation rate. Previous research has clearly shown a deficit in patient knowledge regarding RCTs during enrollment [14, 33]. However, giving particular information about the sponsoring institution may lead surrogates to feel the research is in the best interests of the institution versus the best interests of current or future patients. This approach may seem less personal to the surrogates and perhaps leads to the lower likelihood of participation.

A few limitations of this study are noted. First, a small yet clinically significant sample was used to test hypotheses. Second, the applicability of results to other surrogates may be limited given parents in the PICU are significantly more stressed and more prone to symptoms of posttraumatic stress than parents with children on other hospital services [32]. Nevertheless, this is an important population to study, particularly because of the unique and stressful circumstances of the PICU. Third, there may be other relevant factors that may affect the decision-making process which were not addressed in the study; however, the goal was to address particular factors that would be relevant across sites and populations. Finally, the education level and SES measures were likely not sufficiently sensitive as categories were notably broad. Additional control of these variables may be informative in future studies. The study also showcases strengths as multiple informants were used whenever possible, nonmodifiable participant characteristics were considered as relevant covariates, and findings represent a contribution to a clinically significant body of literature surrounding decision making in a unique environment.

## 5. Conclusion

The critical role that physicians play in medical decision making is well known [16, 17]. Physicians undoubtedly play a role in treatment decisions made by their patients' families in the pediatric intensive care unit. However, this influence is reduced in the context of recruitment in clinical trials in the PICU, which is consistent with ethical standards related to the consenting process. In addition, type of HCP is not related to the decision making process, which also highlights the equity in the decision-making process and adherence to ethical standards. Nevertheless, there are subtle communication factors that play a role in this process. For instance, participants may possibly feel deceived or pressured when the sponsoring institution is mentioned or strong recommendations to participate are given. As any kind of pressure to participate is counter to the informed consent process, such techniques should be strongly discouraged and avoided. The current study indicates that subtleties of HCP communication merit further investigation regarding their influence on participation in a pediatric RCT. It also suggests that the age of the child is likely a significant factor in this decision making process. The influence of these subtle factors on decision making as well as the impact of direct recommendations should be evaluated in more depth and with additional participants in future studies. Further, the significant HCP influences may not only play a role in the high stress environment of the PICU, but also play a role in other areas of pediatrics. Expansion of the current study to other pediatric populations (e.g., those with chronic illnesses) would represent a significant contribution to the literature related to decision making in RCTs beyond the informed consent process to ensure protection for these vulnerable populations.

## Figures and Tables

**Table 1:** Demographics.

Variable	*n*		%
Female surrogate	70		92.0
Male pediatric Patient	44		57.1
Surrogate race			
Caucasian	28		36.8
African American	44		57.9
Other	4		5.3
Single parent Household	35		48.7
12 or fewer years Surrogate education	31		41.9
Income above 25 K Annually	40		49.3

Variable	Mean	SD	Range

Age of pediatric Patient (years)	5.76	4.84	0–17
Age of surrogate (years)	32.13	8.02	17–59

**Table 2:** Demographic factors associated with decision to participate in the RCT.

Variable	*χ* ^2^	df	*P*	*t*	df	*P*
Surrogate gender	.03	1	>.05			
Surrogate race	.001	1	>.05			
Marital status	2.27	2	>.05			
Surrogate Education level	3.04	2	>.05			
Income level	2.23	4	>.05			
Surrogate age				.80	20.09	>.05
Pediatric patient age				3.00	22.30	<.005∗

Note: df = degrees of freedom, *significant.

**Table 3:** *β* Weights and Chi-Square Results for Logistic Regression Analysis.

Hypothesis	*β*	*χ* ^2^	*P*	Cox & Snell *R* ^2^	Nagelkerke *R* ^2^
(1) Age of Pediatric Patient	−0.19				
HCP Recommendation	−0.001	4.90	>.05	.06	.11

(2) Age of Pediatric Patient	−0.20				
Type of HCP Presenting	−0.40	5.08	>.05	.07	.12

(3) Age of Pediatric Patient	−0.09				
Mention Sponsoring Institution	−2.30	11.05	<.005∗	.14	.14

Note: HCP = Health Care Provider, *significant.
